# Association of Axillary Lymph Node Evaluation With Survival in Women Aged 70 Years or Older With Breast Cancer

**DOI:** 10.3389/fonc.2020.596545

**Published:** 2021-01-28

**Authors:** Shi-Ping Luo, Jie Zhang, Qi-Sen Wu, Yu-Xiang Lin, Chuan-Gui Song

**Affiliations:** ^1^ Department of Breast Surgery, Fujian Medical University Union Hospital, Fuzhou, China; ^2^ Department of General Surgery, Fujian Medical University Union Hospital, Fuzhou, China; ^3^ Department of Orthopedics, Fujian Medical University Union Hospital, Fuzhou, China

**Keywords:** sentinel lymph node biopsy, axillary lymph node dissection, elderly breast cancer, propensity score matching, Surveillance Epidemiology and End Results database

## Abstract

**Background:**

Survival in elderly patients undergoing sentinel lymph node biopsy (SLNB) and axillary lymph node dissection (ALND) has not been specifically analyzed. This study aimed to explore the association between different types of axillary lymph node (ALN) evaluations and survival of elderly breast cancer patients.

**Methods:**

A retrospective cohort study was conducted of invasive ductal breast cancer patients 70 years and older in the Surveillance, Epidemiology, and End Results database (2004–2016). Analyses were performed to compare the characteristics and survival outcomes of patients who received surgical lymph node dissection and those who did not. Breast cancer specific survival (BCSS) and overall survival were compared by using Cox proportional hazards regression analysis and propensity score matching (PSM) methods to account for selection bias from covariate imbalance.

**Results:**

Of the 75,950 patients analyzed, patients without ALN evaluation had a significantly worse prognosis, while there was no significant difference for BCSS between using a sentinel lymph node biopsy (SLNB) and an axillary lymph node dissection (ALND) after adjustment for known covariates [adjusted hazard ratio (HR) = 0.991, 95% confidence interval (CI) = 0.925–1.062, *p* = 0.800]. In the stratification analyses after PSM, the ALND did not show a significant BCSS advantage compared with SLNB in any subgroups except for the pN1 stage or above. Furthermore, after PSM of the pN1 stage patients, SLNB was associated with a significantly worse BCSS in hormone receptor negative (HR−) patients (HR = 1.536, 95%CI = 1.213–1.946, *p <* 0.001), but not in the hormone receptor positive (HR+) group (HR = 1.150, 95%CI = 0.986–1.340, *p* = 0.075).

**Conclusion:**

In our study, ALND does not yield superior survival compared with SLNB for elderly patients with pN1 stage HR+ breast cancer. Although our findings are limited by the bias associated with retrospective study design, we believe that in the absence of results from randomized clinical trials, our findings should be considered when recommending the omission of ALND for elderly breast cancer patients.

## Introduction

Since the early 2000s surgical techniques for axillary treatment and staging of patients with primary breast cancer have become less extensive and more focused on minimizing the risk related to surgery (1). Sentinel lymph node biopsy (SLNB) could reduce the side effects of axillary lymph node dissection (ALND) within a certain range of adaptation and provide an equivalent outcome. The National Surgical Adjuvant Breast and Bowel Project (NSABP) B32 trial (2) validated that the usage of SLNB for avoiding ALND in patients with clinically node-negative (cN0) breast cancer had no impact on prognosis. The American College of Surgeons Oncology Group (ACOSOG) Z0011 trial (3) eliminated the demand for ALND for breast cancer patients with one or two positive sentinel lymph nodes who were treated with breast conserving surgery (BCS) and whole breast irradiation.

However, there are no clinical studies specifically for elderly breast cancer patients previously, and the evidence of optimal axillary lymph node evaluation is limited. In 2012, the International Society of Geriatric Oncology (SIOG) and European Society of Breast Cancer Specialists (EUSOMA) (4) updated their recommendations regarding elderly breast cancer (EBC) patients. It was proposed that elderly patients with cN0 breast cancer could be exempted from axillary lymph node evaluation. Since no survival improvement with ALND was identified in relevant studies (5, 6), the Society of Surgical Oncology Choosing Wisely Guidelines recommended in 2016 that surgeons “do not routinely use sentinel node biopsy in clinically node-negative women ≥70 years of age with hormone receptor-positive (HR+) invasive breast cancer”. This recommendation aroused extensive discussion (7–9) about whether cN0 elderly breast cancer patients can be exempted from axillary lymph node evaluation. No clinical studies have yet been conducted to investigate the difference in survival between SLNB and ALND in elderly breast cancer patients.

A sentinel lymph node biopsy is minimally invasive compared with axillary lymph node dissection, with the risk of lymphedema being only 3–7% for SLNB while it is 15–20% for ALND (10). In the era of precision medicine, our study aimed to explore the association between different types of axillary lymph node evaluations with survival and provide new insight into axillary management for elderly breast cancer patients.

## Methods

### Data Source and Study Population

Women 70 years and older with invasive ductal breast cancer diagnosed between January 2004 and December 2016 were retrieved from the Surveillance, Epidemiology, and End Results (SEER) Program (Nov 2018 Submission). We utilized the SEER*Stat version 8.3.6 to extract the target population’s information. Patients with missing or unknown T-, N-, M-stage, grade, estrogen receptor (ER) status, progesterone receptor (PR) status, number of lymph nodes (LNs) removed, surgery type, or survival data were excluded from this study, so were patients with metastatic disease (**Supplemental Figure 1**). The data elements included patient characteristics, cancer staging, type and timing of first course of treatment, as well as survival outcome information. The SEER database did not specify the axillary surgery type as ALND or SLNB. Therefore, we use the number of nodes examined as an alternative in this study. According to the definition of ALND, which was set as a standard by the American Joint Commission on Cancer (AJCC), ALND should involve at least six lymph nodes. Hence, we used five examined lymph nodes as the cut-off value for SLNB and ALND. Patients with five or fewer lymph nodes examined were categorized as having received SLNB, while patients with six or more nodes examined were categorized as having undergone ALND (11, 12).Those with 0 to 5 positive regional lymph nodes were included into this study. Patients with more than five positive lymph nodes, who might have a worse prognosis, would be directly assigned into the ALND group within the classification rules.

For the general population, the study groups were defined as those who underwent surgical LN evaluation, including SLNB (fewer than six lymph nodes examined) and ALND (six or more lymph nodes examined) and aim to identify the survival differences among the three groups, then to obtain relevant information on whether axillary assessment could be exempted. For the pathological stage N1 cohort, the survival of SLNB and ALND patients was further evaluated and compared in order to get information on the conditions under which ALND can be avoided when a small number of lymph nodes are positive. The primary endpoint of this study was breast cancer specific survival (BCSS).

### Statistical Analysis

Patient characteristics are summarized with *N* (%) of inclusion categorical variables and *mean* (SD) of the number of examined nodes and survival time. Associations between axillary surgery modality, patient demographics, and clinical pathological characteristics were assessed using the Pearson χ^2^ or Fisher’s exact test and the Wilcoxon rank sum test for continuous variables. The Kaplan–Meier method was applied to generate unadjusted survival curves, while the log-rank test was used to assess the differences. Univariable and multivariable Cox proportional hazards regression analysis was conducted to estimate the association between different types of axillary lymph node evaluations and survival after adjusting for exploratory variables that were shown to have a significant effect on survival.

To avoid the impact of the different characteristics between the two study groups (SLNB group *vs.* ALND group), we adopted the 1:1 nearest neighbor propensity score matching (PSM) method to eliminate the imbalance. Within the matched patient groups, we assessed survival outcomes of different axillary surgery effects with stratification analyses and explored the different effects in patient-, tumor-, and treatment-level subgroups. Kaplan–Meier estimators were calculated for each group and were compared by using the log-rank test.

All tests were two-sided, and a *p* value less than 0.05 was considered to be statistically significant. All statistical analyses were performed using IBM SPSS software version 24.0 (IBM Corp., Armonk, USA) and R version 3.6.2 (The R Project for Statistical Computing, Vienna, Austria).

## Results

### Basic Characteristics and Survival Analyses of the Overall Population

A total of 75,950 eligible elderly breast cancer patients (the median follow-up time was 64 months) were included in this retrospective analysis, of whom 46,253 (60.9%) underwent SLNB, 18,346 (24.2%) underwent ALND, and 11,351 (14.9%) did not receive LN evaluation (the No group) with the median follow-up time of 58, 84 and 58 months respectively. Patient characteristics are listed in **Supplemental Table 1**. Elderly patients had more Luminal-type breast cancer, and among them the ER positive-type made up 84.2% and among the PR positive-type 72.9%. There were fewer human epidermal growth factor receptor-2 (HER2) positive patients (7.1%) than HER2 negative patients (52.1%). Fewer patients received LN evaluation in the older age groups. The proportion of elderly breast cancer patients undergoing SLNB who were diagnosed after 2010 (52.8%) was higher than that in 2004–2009 (34.8%). The patients in the SLNB group received more lumpectomies (75.3%) and more radiotherapy (55.1%), while the ALND group had more mastectomies (58.3%).

**Figure 1:**
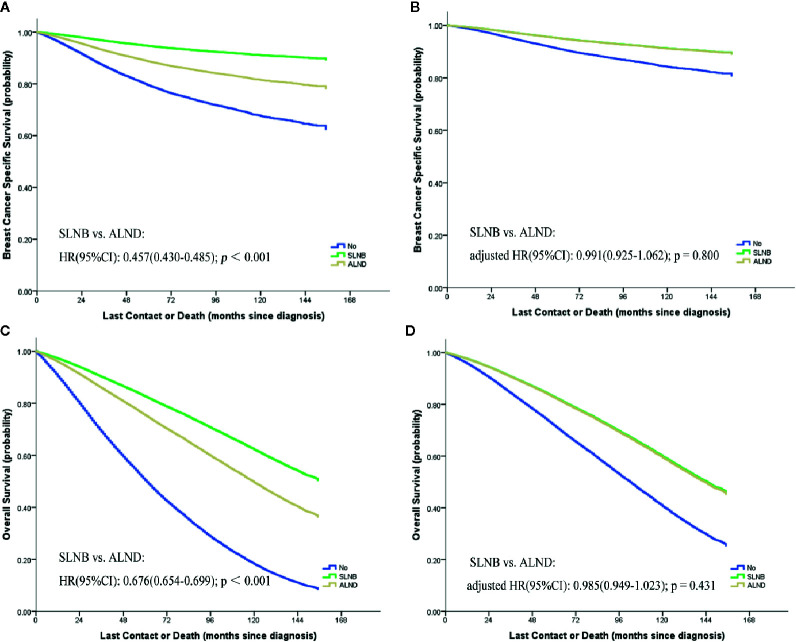
Kaplan–Meier curves [**(A)** breast cancer specific survival; **(C)** overall survival] and the survival curves of adjusted by other prognostic factors [**(B)** breast cancer specific survival; **(D)** overall survival] stratified by different types of axillary lymph node evaluations.

The results of univariable and multivariable Cox proportional hazard regression analyses are shown in **Supplemental Table 2**. It was found that age, race, marital status, grade, T stage, N stage, ER, PR, HER2 status, and different types of adjuvant treatments were independent prognostic factors for elderly breast cancer patients. Survival curves stratified by different types of axillary lymph node evaluations before matching are reported in **Figure 1**. In the univariate analysis, the SLNB group had a better BCSS performance than the ALND group (HR = 0.457, 95%CI = 0.430–0.485, *p <* 0.001). However, after adjusting for the other prognostic factors, there were no significant differences in BCSS between the two groups (SLNB group *vs.* ALND group: adjusted HR = 0.991, 95%CI = 0.925–1.062, *p* = 0.800), while the cohort without a LN evaluation had the worst prognosis.

### Stratification Analyses of the Matched SLNB and ALND Groups

**Figure 2:**
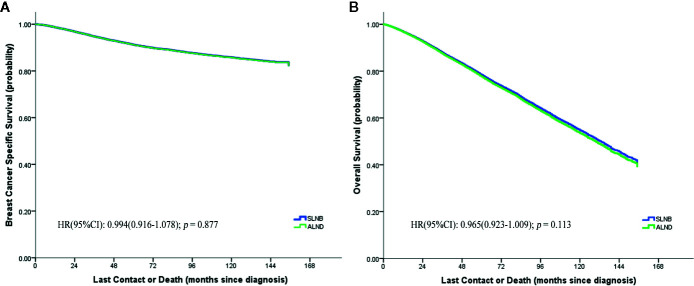
Breast cancer specific **(A)** and overall **(B)** survival curves of the two matched study groups.

We performed a 1:1 PSM with maximum allowed differences of ±0.5% for propensity scores on the SLNB and ALND groups (**Supplemental Figure 2**). Relevant results of the matched stratification analyses are displayed in **Table 1**. In the matched groups, the SLNB group and the ALND group did not show significant BCSS differences (HR 0.994, 95%CI = 0.916–1.078, *p* = 0.877) (**Figure 2A**) and OS differences (HR = 0.965, 95% CI = 0.923–1.009, *p* = 0.113) (**Figure 2B**). Similar results were observed in the different patient, tumor, and treatment subgroups, except in the Grade 1, T4 stage, and different N stage subgroups. In the pN0 stage subgroup, the SLNB group had a better breast cancer prognosis. On the contrary, the prognosis of the ALND group was better with N1 stage and above.

**Table 1:** The matched stratification analyses of breast cancer specific survival (BCSS).

	SLNB group			ALND group			BCSS	
Variables	No. of patients	No. of events	Survival Rates	No. of patients	No. of events	Survival Rates	HR (95%CI)	*P value*
**Total**	13,246	1141	91.4%	13,246	1,162	91.20%	0.994(0.916–1.078)	0.877
**Year at diagnosis**								
2004-2009	6,847	781	88.6%	6,588	804	87.8%	0.945(0.856–1.042)	0.258
2010-2016	6,399	360	94.4%	6,658	358	94.6%	1.115(0.963-1.291)	0.145
**Age**								
70–74	5,270	310	94.1%	5,358	343	93.6%	0.945(0.810–1.101)	0.467
75–79	3,939	325	91.7%	3,942	347	91.2%	0.927(0.797–1.079)	0.329
80–84	2,553	281	89.0%	2,576	282	89.1%	1.013(0.859–1.195)	0.875
85+	1,484	225	84.8%	1,370	190	86.1%	1.114(0.918–1.352)	0.273
**Race**								
White	1,0843	968	91.1%	11,124	953	91.4%	1.054(0.964–1.153)	0.246
Black	1,125	112	90.9%	1,220	142	88.4%	0.843(0.658–1.080)	0.176
Other	1,278	61	95.2%	902	67	92.6%	0.675(0.477–0.954)	0.026
**Marital**								
Married	5,526	412	92.5%	5,648	424	92.5%	1.019(0.890–1.167)	0.782
Single	7,130	681	90.4%	7,042	694	90.1%	0.978(0.880–1.087)	0.675
Unknown	590	48	91.9%	556	44	92.1%	0.950(0.630–1.431)	0.806
**Laterality**								
Right	6,397	518	91.9%	6,562	545	91.7%	0.981(0.870–1.106)	0.755
Left	6,849	623	90.9%	6,684	617	90.8%	1.003(0.897–1.121)	0.959
**Grade**								
I	2,691	71	97.4%	2,695	105	96.1%	0.683(0.506–0.923)	**0.013**
II	5,951	386	93.5%	5,954	404	93.2%	0.999(0.869–1.149)	0.990
III	4,604	684	85.1%	4,597	653	85.8%	1.027(0.922–1.143)	0.633
**T Stage**								
T1	8,171	406	95.0%	8,108	421	94.8%	0.956(0.834–1.096)	0.517
T2	4,367	552	87.4%	4,366	589	86.5%	0.977(0.870–1.097)	0.690
T3	396	88	77.8%	424	79	81.4%	1.206(0.890–1.635)	0.226
T4	312	95	69.6%	348	73	79.0%	1.716(1.264–2.331)	**0.001**
**N Stage**								
N0	8,771	550	93.7%	8,709	644	92.6%	0.819(0.731–0.918)	**0.001**
N1	4,268	521	87.8%	4,242	472	88.9%	1.243(1.097–1.408)	**0.001**
N2	183	61	66.7%	267	44	83.5%	2.886(1.950–4.271)	**<0.001**
N3	24	9	62.5%	28	2	92.9%	5.465(1.180–25.307)	**0.030**
**Type of Surgery**								
No	14	4	71.4%	16	4	75.0%	1.019(0.253–4.103)	0.979
BCS	6,579	417	93.7%	6,662	454	93.2%	0.950(0.832–1.085)	0.451
Mastectomy	6,653	720	89.2%	6,568	704	89.3%	1.013(0.913–1.124)	0.812
**Radiation**								
Yes	5,567	372	93.3%	5,432	385	92.9%	0.957(0.830–1.104)	0.546
No/Refused	7,679	769	90.0%	7,814	777	90.1%	1.019(0.922–1.126)	0.715
**Chemotherapy**								
Yes	2,651	277	89.6%	2,746	251	90.9%	1.206(1.017–1.431)	**0.031**
No/Unknown	10,595	864	91.8%	10,500	911	91.3%	0.943(0.859–1.034)	0.213
**ER Status**								
Positive	10,724	729	93.2%	10,730	766	92.9%	0.976(0.882–1.081)	0.645
Negative	2,522	412	83.7%	2,516	396	84.3%	1.005(0.876–1.154)	0.941
**PR Status**								
Positive	9,084	535	94.1%	9,202	604	93.4%	0.920(0.819–1.034)	0.163
Negative	4,162	606	85.4%	4,044	558	86.2%	1.041(0.928–1.167)	0.497
**HER2 Status**								
Positive	916	71	92.2%	964	61	93.7%	1.347(0.956–1.897)	0.088
Negative	5,331	281	94.7%	5,527	285	94.8%	1.088(0.923–1.284)	0.314
Borderline	152	8	94.7%	167	12	92.8%	0.677(0.277–1.657)	0.393
Not 2010+	6,847	781	88.6%	6,588	804	87.8%	0.945(0.856–1.042)	0.258

### Exploratory Analyses of pN1 Stage Matched Groups

**Figure 3:**
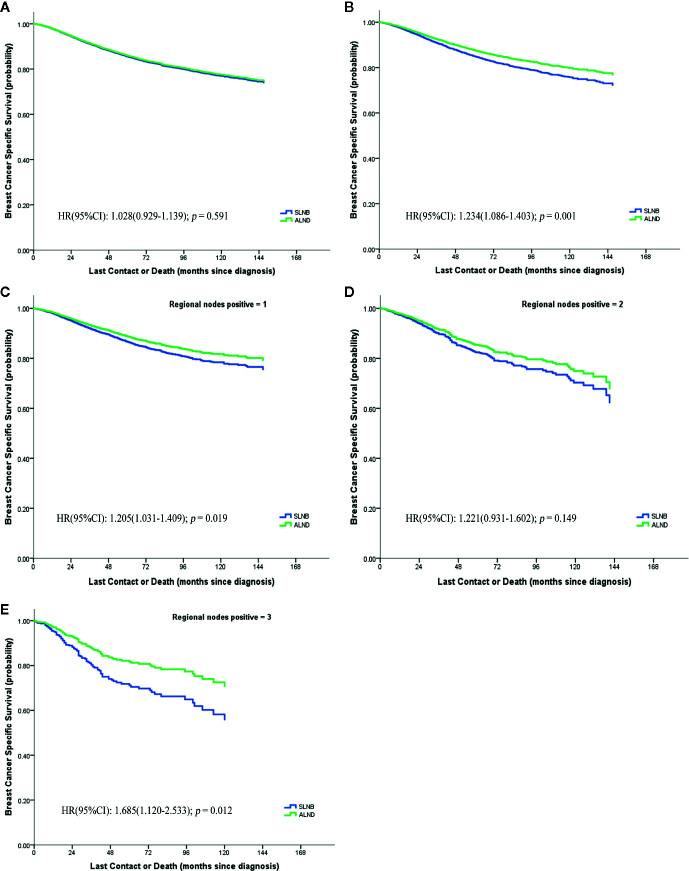
Breast cancer specific survival of pN1 stage, **(A)** before matching; **(B)** after matching; **(C–E)** the numbers of positive lymph node respectively are 1, 2, 3.

For exploratory analyses of the pN1 stage cohort, elderly breast cancer patients treated with SLNB were matched 1:1 to patients from the ALND group (**Supplemental Figure 3**); the baseline characteristics before and after matching are listed in **Supplemental Table 3**. Regardless of matching or not, all the variables were identified to be significantly associated with BCSS except for the marital status and HER2 status (**Table 2**). Kaplan–Meier curves of the whole cohort in the two axillary surgery groups revealed no significant differences (HR = 0.972, 95%CI = 0.878–1.077, *p* = 0.591) **(** **Figure 3A**). However, after adjustments using other prognostic factors, the risk of death in the ALND group was significantly lower than in the SLNB group, both before and after matching cohorts (**Table 2**; **Figure 3B**).

**Table 2:** Multivariate analysis by Cox proportional hazard model in before and after matching pN1 stage cohorts.

Variables		Before Matching		After Matching	
		HR (95%CI)	*P* value	HR (95%CI)	*P* value
**Age**	**70–74**	Ref		Ref	
	**75–79**	1.225(1.074**–**1.396)	**0.002**	1.215(1.014**–**1.454)	**0.034**
	**80–84**	1.450(1.258**–**1.672)	**<0.001**	1.422(1.174**–**1.723)	**<0.001**
	**85+**	1.716(1.463**–**2.014)	**<0.001**	1.729(1.408**–**2.124)	**<0.001**
**Race**	**White**	Ref		Ref	
	**Black**	1.139(0.981**–**1.323)	0.088	1.112(0.898**–**1.377)	0.331
	**Other**	0.680(0.534**–**0.865)	**0.002**	0.635(0.456**–**0.885)	**0.007**
**Marital**	**Married**	Ref		Ref	
	**Single**	1.017(0.914**–**1.132)	0.759	1.050(0.911**–**1.211)	0.502
	**Unknown**	0.870(0.659**–**1.149)	0.327	0.867(0.598**–**1.257)	0.451
**Grade**	**I**	Ref		Ref	
	**II**	1.975(1.560**–**2.500)	**<0.001**	1.897(1.426**–**2.522)	**<0.001**
	**III**	3.112(2.450**–**3.951)	**<0.001**	3.035(2.269**–**4.061)	**<0.001**
**T Stage**	**T1**	Ref		Ref	
	**T2**	1.900(1.686**–**2.141)	**<0.001**	1.900(1.629**–**2.216)	**<0.001**
	**T3**	3.214(2.639**–**3.914)	**<0.001**	2.905(2.204**–**3.829)	**<0.001**
	**T4**	3.413(2.799**–**4.163)	**<0.001**	2.922(2.240**–**3.810)	**<0.001**
**The Number of Positive LN**	**1**	Ref		Ref	
	**2**	1.273(1.133**–**1.430)	**<0.001**	1.224(1.044**–**1.433)	**0.012**
	**3**	1.473(1.281**–**1.694)	**<0.001**	1.539(1.234**–**1.920)	**<0.001**
**Type of Surgery**	**No**	Ref		Ref	
	**BCS**	0.280(0.203**–**0.386)	**<0.001**	0.127(0.047**–**0.342)	**<0.001**
	**Mastectomy**	0.295(0.217**–**0.403)	**<0.001**	0.135(0.050**–**0.364)	**<0.001**
**Type of Axillary Surgery**	**SLNB**	**Ref**		**Ref**	
	**ALND**	**0.763(0.682–0.853)**	**<0.001**	**0.781(0.686–0.889)**	**<0.001**
**Radiation**	**Yes**	Ref		Ref	
	**No**	1.464(1.299**–**1.651)	**<0.001**	1.427(1.220**–**1.668)	**<0.001**
**Chemotherapy**	**Yes**	Ref		Ref	
	**No**	1.457(1.292**–**1.645)	**<0.001**	1.541(1.302**–**1.824)	**<0.001**
**ER Status**	**Positive**	Ref		Ref	
	**Negative**	1.559(1.352**–**1.798)	**<0.001**	1.404(1.162**–**1.698)	**<0.001**
**PR Status**	**Positive**	Ref		Ref	
	**Negative**	1.644(1.437**–**1.880)	**<0.001**	1.757(1.477**–**2.089)	**<0.001**
**HER2 Status**	**Positive**	Ref		Ref	
	**Negative**	1.090(0.880**–**1.348)	0.430	0.989(0.763**–**1.283)	0.935
	**Borderline**	0.938(0.546**–**1.612)	0.816	1.085(0.574**–**2.050)	0.802
	**Not 2010+**	1.247(1.018**–**1.528)	0.033	1.061(0.824**–**1.365)	0.648

Bold P values mean that the difference is statistically significant.

**Figure 4:**
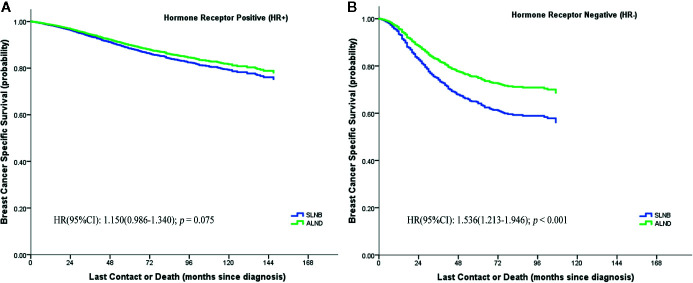
Breast cancer specific survival of hormone receptor positive (HR+) **(A)** and hormone receptor negative (HR−) **(B)** stratified by SLNB and ALND in the matching pN1 stage patients.

We further evaluated whether the BCSS advantage of ALND still existed when considering different numbers of positive lymph nodes or different hormone receptor status (**Figures 3C–E**). It demonstrates that the SLNB group still showed a survival disadvantage in BCSS compared to the ALND group even though there was only one positive lymph node (HR = 1.205, 95%CI = 1.031–1.409, *p* = 0.019) (**Figure 3C**). Moreover, the survival differences between the two groups was also affected by the hormone receptor status. In the hormone receptor positive (HR+) subgroup the ALND group patients no longer had an absolute BCSS advantage (HR = 1.150, 95%CI = 0.986–1.340, p = 0.075) (**Figure 4A**). Whereas, the hormone receptor negative (HR−) subgroup had similar outcomes (SLNB group *vs.* ALND group: HR = 1,536, 95%CI = 1.213–1.946, *p <* 0.001) (**Figure 4B**).

### Further Exploratory Analysis in Number of Positive Lymph Nodes and Hormone Receptor Status

**Figure 5:**
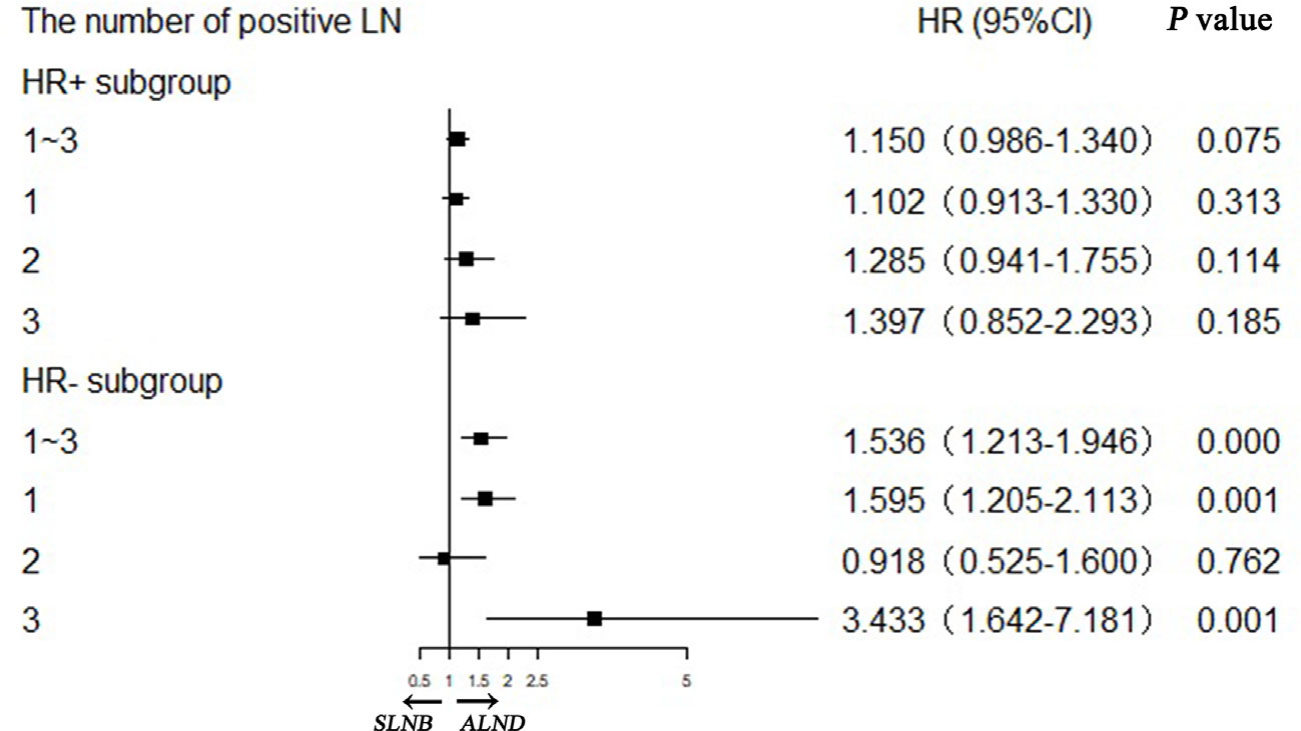
Stratification analyses of hormone receptor positive (HR+) and hormone receptor negative (HR−) subgroups with different positive lymph node numbers in matching pN1 stage patients.

We confirmed that the baseline characteristics of the HR+ and HR− subgroups were comparable in the matched SLNB group and ALND group (**Supplemental Table 4**). **Figure 5** shows the hazard ratios (HRs) of the SLNB group *versus* the ALND group on the basis of various combinations of hormone receptor status and number of positive lymph nodes. In the HR+ subgroup the SLNB groups were comparable with the ALND groups in BCSS performance regardless of the number of positive lymph node. While for the HR− subgroup, the BCSS of SLNB group was worse than that of the ALND group although only one lymph node was positive, and the SLNB group had worse survival when there were more positive lymph nodes.

## Discussion

More than 30% of breast cancers are diagnosed in patients older than 70 years old (13, 14). By now, the average life expectancy of women over the age of 65 is 86.6 years, with one in four women achieving an age above 90 years old (15). Our study is of particular importance in light of the aging population and serves as a reference since there is a lack of randomized data to guide clinical decision-making. The most common manifestation of breast cancer in elderly patients is a higher grade and HR-positive invasive ductal carcinoma (**Table 1**). Some studies have also indicated that the incidence of ER+ breast cancer increased and that of HER2 decreased with age (14, 16, 17).

Previously published high-quality prospective studies of axillary treatments did not focus on elderly patients exclusively (2, 3, 18, 19). These studies paid more attention to whether axillary evaluation could be omitted (5–8, 20). The International Society of Geriatric Oncology (SIOG) and European Society of Breast Cancer Specialists (EUSOMA) in 2012 (4) and the Society of Surgical Oncology of the Choosing Wisely campaign in 2016 recommended that elderly breast cancer patients could be exempted from axillary lymph node evaluation when it was clinically determined that axillary lymph nodes were negative (5, 6). A subsequent meta-analysis composed of two randomized controlled trials involving 692 patients found that omission of axillary evaluation would not result in significant difference of overall breast cancer specific mortality (21).

In our study we demonstrated that, after adjustment by other factors, axillary lymph node surgery (both SLNB and ALND) raised the breast cancer-specific survival by more than 40% compared to patients who did not receive lymph node assessment (**Table 2**). Similarly, Chagpar et al. (7) revealed that after controlling for tumor size, grade, patient age, comorbidities, and treatment factors, patients who did not have LN evaluation had a worse survival compared with those who had axillary evaluation. It was also indicated that axillary surgery was associated with higher rates of adjuvant therapy and improved overall survival for elderly cN0 breast cancer patients in a study from Tamirisa et al. (22). Lymph node evaluation was shown to provide important information for determining their adjuvant therapy options (7).

It is well known that SLNB is minimally invasive, with a 2–7% risk of upper extremity lymphedema, in comparison with the 15–20% risk associated with ALND (10, 23). Therefore, in the social context of population aging and precision medicine, it is necessary and imperative to identify whether elderly patients need ALND or not. To the best of our knowledge, this is the largest cohort that has been evaluated to compare SLNB and ALND in elderly breast cancer patients. We performed PSM analyses to address the limitations of a retrospective study from a large SEER sample of patients who underwent axillary surgery. After reliable Cox regression analyses and matched stratification analyses, SLNB did not imply higher breast cancer specific mortality among the cohort, in both subgroups with or without other kind of treatments and regardless of the ER, PR, and HER2 status.

We were concerned that the survival of SLNB group patients is concentrated in the stage N0 patients; in the stage N1 and above patients ALND still needs to be selected. A meta-analysis based on four trials showed no significant differences in OS and DFS between ALND and regional nodal irradiation (RNI) in short- or long-term outcomes (24). Hence, RNI may be an alternative treatment for adjuvant management of the axilla in selected patients, and an optimal radiation strategy approach for elderly patients warrants further study. However, it is undeniable that local control of the axilla isõ still important in the treatment of elderly breast cancer patients.

Our exploratory analyses for the stage N1 cohort detected that with HR+ breast cancer in elderly patients with 1 to 3 positive lymph nodes, you could omit further lymph node dissection: True in both the with and without radiation subgroups. The HR− patients still required ALND even when there was only one positive lymph node. Some studies have concluded that the adjuvant therapy strategies for HR+ elderly breast cancer should only be followed by endocrine therapy, and the axillary lymph node dissection can be avoided (5, 20). At present, the guidelines for breast cancer therapy recommend that the standard adjuvant endocrine therapy for postmenopausal patients is five years of aromatase inhibitor (AI). And for patients at high risk, a prolonged AI treatment can reduce the risk of relapse (25–28). In elderly patients with HR+ breast cancer, endocrine therapy plays an important role in the adjuvant therapy. Therefore, we hypothesize from our observations that the method of performing intensive endocrine therapy is more important than local treatment in the case of sentinel lymph nodes.

Inevitably, there are several limitations related to the design and data source in our study. Firstly, the number of examined recorded in the SEER database is the final total removed number, and unfortunately, we cannot determine the exact procedure of the axilla surgery. Even though the analyses based on PSM could effectively reduce the effects of the observed confounding factors, it cannot address unobserved confounding factors, nor the unavoidably cases lost. Secondly, the data about endocrine therapy in the SEER database is inaccessible despite the importance in adjuvant treatment of HR+ breast cancer, which makes the analyses of adjuvant therapy for elderly breast cancer incomplete. Thirdly, locoregional recurrence or disease-free survival is not included in the SEER database, and this precludes assessment of these end points. Lastly, it is unfortunate that cases receiving neoadjuvant chemotherapy could not be identified in the SEER database, which may lead to changes in axillary management.

To summarize, our findings suggest that ALND can be omitted in elderly patients with pN1 stage HR+ breast cancer. This study is the first to use a large number of cases of elderly patients for evaluation of the relative effectiveness between SLNB and ALND with BCSS as the primary endpoint. Although our findings are limited by the bias associated with retrospective study design, we believe that in the absence of randomized clinical trials, our findings should be considered when recommending the omission of ALND for elderly breast cancer patients. However, we still need further accurate prospective randomized studies to optimize patient selection for the omission of ALND.

## Data Availability Statement

Publicly available datasets were analyzed in this study. This data can be found here: https://seer.cancer.gov/.

## Author Contributions

S-PL and JZ conceptualized and designed the study. S-PL, JZ, and Q-SW developed the methodology. S-PL, JZ, and Y-XL took part in the acquisition, analysis, and interpretation of the data. S-PL, JZ, and C-GS wrote, reviewed, and/or revised the manuscript. C-GS and JZ supervised the study. All authors contributed to the article and approved the submitted version.

## Funding

This study was funded by National Natural Science Foundation of China (81672817).

## Conflict of Interest

The authors declare that the research was conducted in the absence of any commercial or financial relationships that could be construed as a potential conflict of interest.

## Supplementary Material

Supplementary Figure 1 Flow diagram for identifying eligible elderly patients (≥70 years old) with breast cancer.

Supplementary Figure 2 The histogram **(A)** and dot plots **(B)** of before and after propensity score matching on SLNB group and ALND group of the total cohort.

Supplementary Figure 3 The histogram **(A)** and dot plots **(B)** of before and after propensity score matching on SLNB group and ALND group of the pN1 stage cohort.
