# Supplementary figures and images for: Association of Axillary Lymph Node Evaluation With Survival in Women Aged 70 Years or Older With Breast Cancer
Source: Front Oncol. 2021 Jan 28;10:596545. doi: 10.3389/fonc.2020.596545 (PMC7877252; doi:10.3389/fonc.2020.596545)

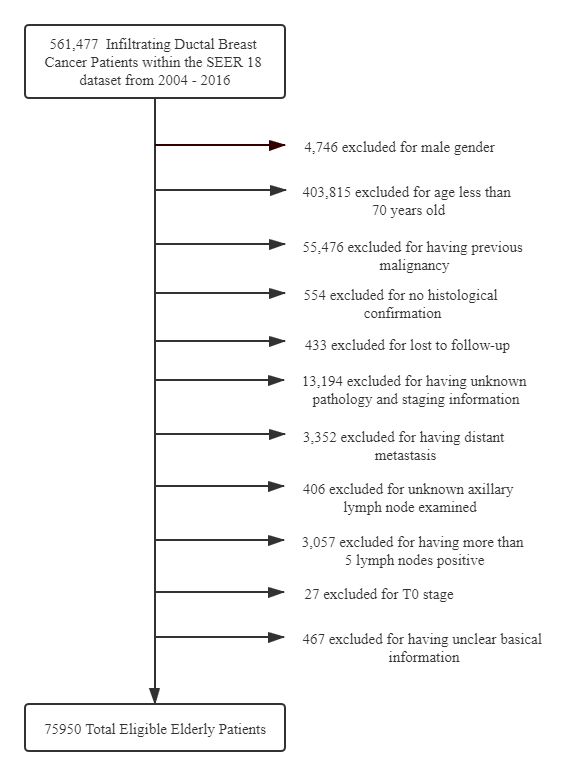

Supplement: Supplementary Figure 1 — Flow diagram for identifying eligible elderly patients (≥70 years old) with breast cancer. [file Image_1.png]

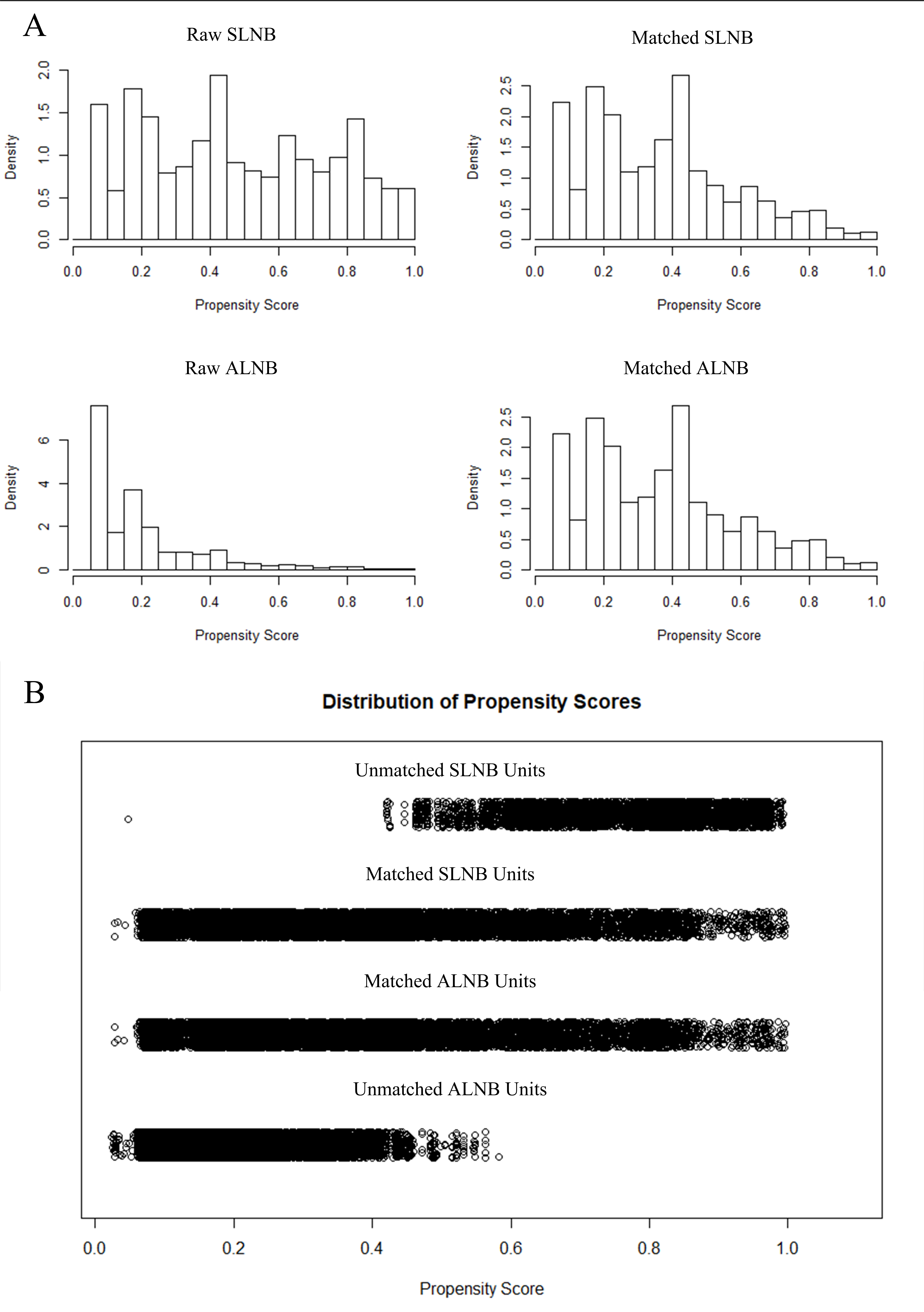

Supplement: Supplementary Figure 2 — The histogram (A) and dot plots (B) of before and after propensity score matching on SLNB group and ALND group of the total cohort. [file Image_2.png]

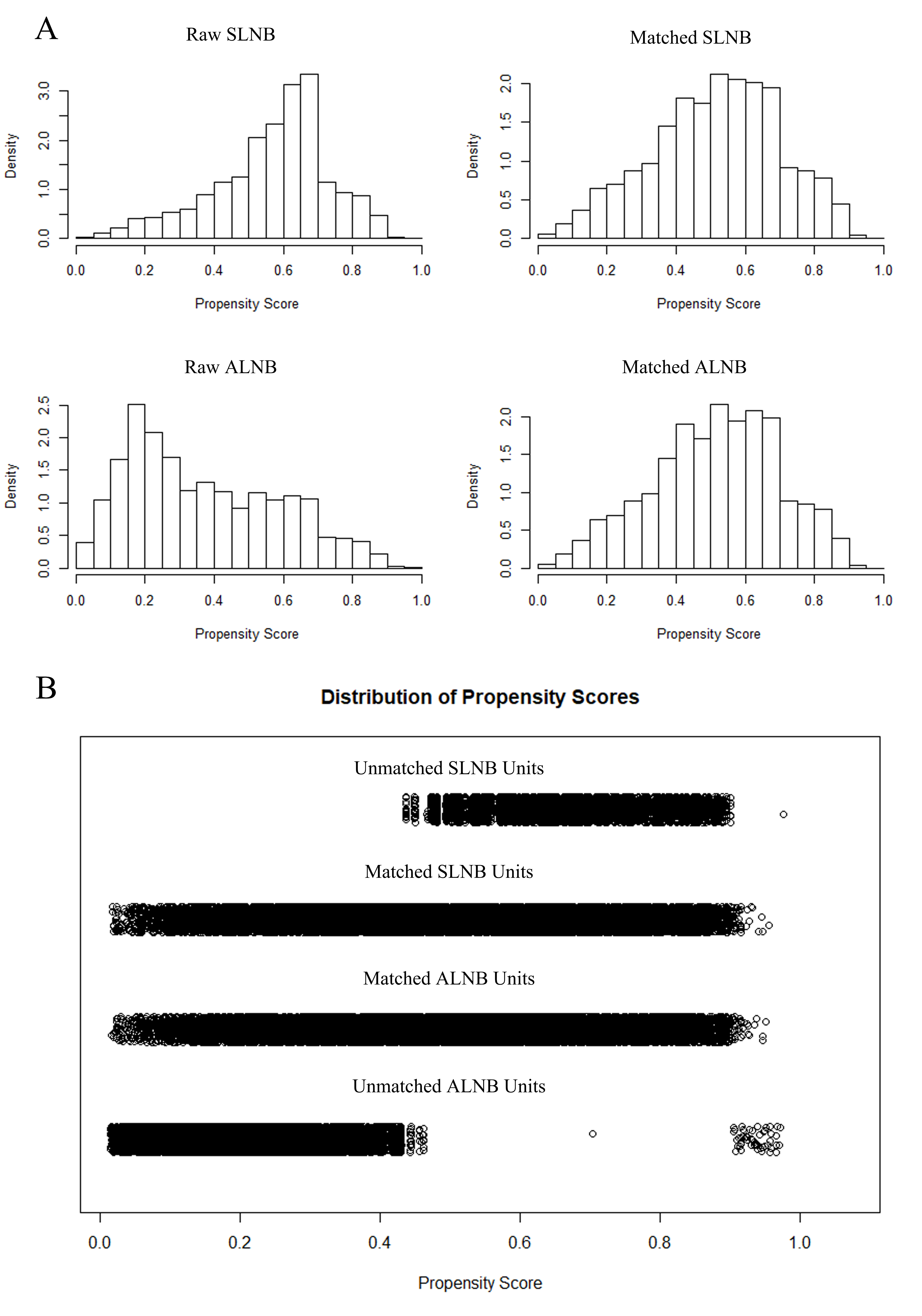

Supplement: Supplementary Figure 3 — The histogram (A) and dot plots (B) of before and after propensity score matching on SLNB group and ALND group of the pN1 stage cohort. [file Image_3.png]
