# Supplementary material for: Association of Axillary Lymph Node Evaluation With Survival in Women Aged 70 Years or Older With Breast Cancer
Source: Front Oncol. 2021 Jan 28;10:596545. doi: 10.3389/fonc.2020.596545 (PMC7877252; doi:10.3389/fonc.2020.596545)
Supplement: Supplementary file 4 [file Table_1.doc]

**Supplemental Table 1.** Demographic and clinicopathological characteristics of patients, stratified by axillary lymph node evaluation type

| **Characteristics** | **Total** | **No** ^a^ | **SLNB** | **ALND** | ***P* value** |
| --- | --- | --- | --- | --- | --- |
| **N** | 75950 | 11351 | 46253 | 18346 |  |
| **Regional nodes examined** ^b^ | 4.34(5.39) | 0.00(0.00) | 2.28(1.24) | 12.21(5.66) | <0.001 |
| **Year at diagnosis** |  |  |  |  | <0.001 |
| 2004-2009 | 29851(39.3) | 4089(36.0) | 16073(34.8) | 9689(52.8) |  |
| 2010-2016 | 46099(60.7) | 7262(64.0) | 30180(65.2) | 8657(47.2) |  |
| **Age** |  |  |  |  | <0.001 |
| 70-74 | 28824(38.0) | 1586(14.0) | 19826(42.9) | 7412(40.4) |  |
| 75-79 | 21308(28.1) | 1989(17.5) | 13860(30.0) | 5459(29.8) |  |
| 80-84 | 14757(19.4) | 2855(25.2) | 8394(18.1) | 3508(19.1) |  |
| 85+ | 11061(14.6) | 4921(43.4) | 4173(9.0) | 1967(10.7) |  |
| **Race** |  |  |  |  | <0.001 |
| White | 64302(84.7) | 9546(84.1) | 39541(85.5) | 15215(82.9) |  |
| Black | 6361(8.4) | 1123(9.9) | 3391(7.3) | 1847(10.1) |  |
| Other ^c^ | 5287(7.0) | 682(6.0) | 3321(7.2) | 1284(7.0） |  |
| **Marital** |  |  |  |  | <0.001 |
| Married | 31640(41.7) | 3031(26.7) | 21093(45.6) | 7516(41.0) |  |
| Single ^d^ | 40906(53.9) | 7613(67.1) | 23189(50.1) | 10104(55.1) |  |
| Unknown | 3404(4.5) | 707(6.2) | 1971(4.3) | 726(4.0) |  |
| **Laterality** |  |  |  |  | 0.54 |
| Right | 36955(48.5) | 5501(48.5) | 22463(48.6) | 8891(49.0) |  |
| Left | 38995(51.3) | 5850(51.5) | 23790(51.4) | 9355(51.0) |  |
| **Grade** |  |  |  |  | <0.001 |
| I | 18980(25.0) | 2879(25.4) | 12943(28.0) | 3158(17.2) |  |
| II | 34733(45.7) | 5361(47.2) | 21301(46.1) | 8071(44.0） |  |
| III | 22237(29.3) | 3111(27.4) | 12009(26.0) | 7117(38.8) |  |
| **T Stage** |  |  |  |  | <0.001 |
| T1 | 51681(68.0) | 6560(57.8) | 35413(76.6) | 9708(52.9) |  |
| T2 | 20185(26.6) | 3368(29.7) | 9696(21.0) | 7121(38.8) |  |
| T3 | 2034(2.7) | 518(4.6) | 676(1.5) | 840(4.6) |  |
| T4 | 2050(2.7) | 905(8.0) | 468(1.0) | 677(3.7) |  |
| **N Stage** |  |  |  |  | <0.001 |
| N0 | 59610(78.5) | 10183(89.7) | 40620(87.8) | 8807(48.0) |  |
| N1 | 14270(18.8) | 915(8.1) | 5416(11.7) | 7939(43.3) |  |
| N2 | 1890(2.5) | 167(1.5) | 184(0.4) | 1539(8.4) |  |
| N3 | 180(0.2) | 86(0.8) | 33(0.1) | 61(0.6) |  |
| **Type of Surgery** |  |  |  |  | <0.001 |
| No | 3502(4.6) | 3261(28.7) | 225(0.5) | 16(0.1) |  |
| BCS | 49295(64.9) | 6827(60.1) | 34832(75.3) | 7636(41.6) |  |
| Mastectomy | 23153(30.5) | 1263(11.1) | 11196(24.2) | 10694(58.3) |  |
| **Radiation** |  |  |  |  | <0.001 |
| Yes | 34959(46.0) | 2294(20.2) | 25502(55.1) | 7163(39.0) |  |
| No/Refused | 40991(54.0) | 9057(79.8) | 20751(44.9) | 11183(61.0) |  |
| **Chemotherapy** |  |  |  |  | <0.001 |
| Yes | 11822(15.6) | 756(6.7) | 6119(13.2) | 4947(27.0) |  |
| No/Unknown | 64128(84.4) | 10595(93.3) | 40134(86.8) | 13399(73.0) |  |
| **ER Status** |  |  |  |  | <0.001 |
| Positive | 63936(84.2) | 9754(85.9) | 39666(85.8) | 14516(79.1) |  |
| Negative | 12014(15.8) | 1597(14.1) | 6587(14.2) | 3830(20.9) |  |
| **PR Status** |  |  |  |  | <0.001 |
| Positive | 55375(72.9) | 8422(74.2) | 34656(74.9) | 12297(67.0) |  |
| Negative | 20575(27.1) | 2929(25.8) | 11597(25.1) | 6049(33.0) |  |
| **HER2 Status** |  |  |  |  | <0.001 |
| Positive | 5417(7.1) | 850(7.5) | 3166(6.8) | 1401(7.6) |  |
| Negative | 39588(52.1) | 6175(54.4) | 26390(57.1) | 7023(38.3) |  |
| Borderline | 1094(1.4) | 237(2.1) | 624(1.3) | 233(1.3) |  |
| Not 2010+ | 29851(39.3) | 4089(36.0) | 16073(34.8) | 9689(52.8) |  |

*SLNB* Sentinel lymph node biopsy; *ALND* axillary lymph node dissection; *BCS* breast-conserving surgery; *ER* estrogen receptor; *PR* progesterone receptor; *HER2* human epidermal growth factor receptor 2

^a^ No lymph nodes removed.

^b^ The results here are mean with *SD* (standard deviation).

^c^ Other includes American Indian/Alaskan native, and Asian/ Paciﬁc Islander.

^d^ Single includes divorced, separated, single (never married), unmarried or domestic partner and widowed.
