# Supplementary material for: Association of Axillary Lymph Node Evaluation With Survival in Women Aged 70 Years or Older With Breast Cancer
Source: Front Oncol. 2021 Jan 28;10:596545. doi: 10.3389/fonc.2020.596545 (PMC7877252; doi:10.3389/fonc.2020.596545)
Supplement: Supplementary file 5 [file Table_2.doc]

**Supplemental Table 2.** Cox regression analyses of breast cancer specific death (BCSS)

| **Variables** |  | **Univariate regression** | | **Multivariate regression** | |
| --- | --- | --- | --- | --- | --- |
|  |  | **HR (95%CI)** | ***P* value** | **HR (95%CI)** | ***P* value** |
| **Year at diagnosis** | **2004-2009** | Ref |  |  |  |
|  | **2010-2016** | 0.896(0.847-0.947) | **<0.001** |  |  |
| **Age** | **70-74** | Ref |  | Ref |  |
|  | **75-79** | 1.371(1.277-1.473) | **<0.001** | 1.264(1.175-1.359) | **<0.001** |
|  | **80-84** | 1.921(1.786-2.067) | **<0.001** | 1.519(1.407-1.641) | **<0.001** |
|  | **85+** | 3.514(3.269-3.778) | **<0.001** | 1.875(1.727-2.037) | **<0.001** |
| **Race** | **White** | Ref |  | Ref |  |
|  | **Black** | 1.738(1.609-1.878) | **<0.001** | 1.165(1.077-1.260) | **<0.001** |
|  | **Other** | 0.772(0.686-0.868) | **<0.001** | 0.733(0.652-0.825) | **<0.001** |
| **Marital** | **Married** | Ref |  | Ref |  |
|  | **Single** | 1.698(1.606-1.795) | **<0.001** | 1.149(1.084-1.219) | **<0.001** |
|  | **Unknown** | 1.351(1.181-1.545) | **<0.001** | 0.943(0.823-1.080) | 0.396 |
| **Laterality** | **Right** | Ref |  |  |  |
|  | **Left** | 1.046(0.994-1.101) | 0.083 |  |  |
| **Grade** | **I** | Ref |  | Ref |  |
|  | **II** | 2.402(2.178-2.649) | **<0.001** | 1.659(1.503-1.832) | **<0.001** |
|  | **III** | 6.206(5.647-6.821) | **<0.001** | 2.704(2.441-2.996) | **<0.001** |
| **T Stage** | **T1** | Ref |  | Ref |  |
|  | **T2** | 3.997(3.774-4.233) | **<0.001** | 2.219(2.084-2.363) | **<0.001** |
|  | **T3** | 8.774(7.945-9 .689) | **<0.001** | 3.335(2.994-3.714) | **<0.001** |
|  | **T4** | 13.244(12.118-14.474) | **<0.001** | 3.720(3.352-4.129) | **<0.001** |
| **N Stage** | **N0** | Ref |  | Ref |  |
|  | **N1** | 2.681(2.537-2.834) | **<0.001** | 1.935(1.813-2.065) | **<0.001** |
|  | **N2** | 5.145(4.674-5.663) | **<0.001** | 2.730(2.451-3.040) | **<0.001** |
|  | **N3** | 12.923(10.278-16.250) | **<0.001** | 2.742(2.163-3.476) | **<0.001** |
| **Type of Surgery** | **No** | Ref |  | Ref |  |
|  | **BCS** | 0.084(0.078-0.091) | **<0.001** | 0.353(0.318-0.392) | **<0.001** |
|  | **Mastectomy** | 0.193(0.179-0.208) | **<0.001** | 0.390(0.351-0.434) | **<0.001** |
| **Type of Axillary** | **No** | Ref |  | Ref |  |
| **Surgery** | **SLNB** | **0.238(0.223-0.254)** | **<0.001** | **0.535(0.491-0.583)** | **<0.001** |
|  | **ALND** | **0.522(0.489-0.557)** | **<0.001** | **0.540(0.489-0.596)** | **<0.001** |
| **Radiation** | **Yes** | Ref |  | Ref |  |
|  | **No/Refused** | 2.280(2.156-2.410) | **<0.001** | 1.378(1.291-1.472) | **<0.001** |
| **Chemotherapy** | **Yes** | Ref |  | Ref |  |
|  | **No/Unknown** | 0.604(0.568-0.642) | **<0.001** | 1.303(1.212-1.400) | **<0.001** |
| **ER Status** | **Positive** | Ref |  | Ref |  |
|  | **Negative** | 3.044(2.885-3.212) | **<0.001** | 1.531(1.416-1.654) | **<0.001** |
| **PR Status** | **Positive** | Ref |  | Ref |  |
|  | **Negative** | 2.677(2.544-2.817) | **<0.001** | 1.487(1.384-1.598) | **<0.001** |
| **HER2 Status** | **Positive** | Ref |  | Ref |  |
|  | **Negative** | 0.529(0.475-0.588) | **<0.001** | 0.966(0.867-1.077) | 0.533 |
|  | **Borderline** | 0.898(0.709-1.138) | 0.373 | 1.024(0.807-1.298) | 0.848 |
|  | **Not 2010+** | 0.657(0.593-0.728) | **<0.001** | 1.117(1.005-1.240) | 0.039 |

*HR* hazard ratio, *CI* confidence interval
