# Supplementary material for: Association of Axillary Lymph Node Evaluation With Survival in Women Aged 70 Years or Older With Breast Cancer
Source: Front Oncol. 2021 Jan 28;10:596545. doi: 10.3389/fonc.2020.596545 (PMC7877252; doi:10.3389/fonc.2020.596545)
Supplement: Supplementary file 6 [file Table_3.doc]

**Supplemental Table 3.** Baseline characteristics of before and after matching in **pN1 stage** cohort

| **Characteristics** |  | **Before Matching** | | | **After Matching** | | |
| --- | --- | --- | --- | --- | --- | --- | --- |
|  |  | **SLNB** | **ALND** | ***P* value** | **SLNB** | **ALND** | ***P* value** |
| **N** |  | 5282 | 7681 |  | 3931 | 3931 |  |
| **Age** | **70-74** | 1988 (37.6) | 3090 (40.2) | <0.001 | 1484 (37.8) | 1455 (37.0) | 0.326 |
|  | **75-79** | 1437 (27.2) | 2272 (29.6) |  | 1079 (27.4) | 1139 (29.0) |  |
|  | **80-84** | 1068 (20.2) | 1461 (19.0) |  | 803 (20.4) | 757 (19.3) |  |
|  | **85+** | 789 (14.9) | 858 (11.2) |  | 565 (14.4) | 580 (14.8) |  |
| **Race** | **White** | 4497 (85.1) | 6327 (82.4) | <0.001 | 3317 (84.4) | 3333 (84.8) | 0.843 |
|  | **Black** | 460 (8.7) | 815 (10.6) |  | 352 (9.0) | 348 (8.9) |  |
|  | **Other** | 325 (6.2) | 539 (7.0) |  | 262 (6.7) | 250 (6.4) |  |
| **Marital** | **Married** | 2197 (41.6) | 3113 (40.5) | 0.031 | 1609 (40.9) | 1651 (42.0) | 0.621 |
|  | **Single** | 2842 (53.8) | 4272 (55.6) |  | 2158 (54.9) | 2116 (53.8) |  |
|  | **Unknown** | 243 (4.6) | 296 (3.9) |  | 164 (4.2) | 164 (4.2) |  |
| **Laterality** | **Right** | 2587 (49.0) | 3725 (48.5) | 0.602 | 1921 (48.9) | 1889 (48.1) | 0.484 |
|  | **Left** | 2695 (51.0) | 3956 (51.5) |  | 2010 (51.1) | 2042 (51.9) |  |
| **Grade** | **I** | 1033 (19.6) | 1010 (13.1) | <0.001 | 667 (17.0) | 656 (16.7) | 0.712 |
|  | **II** | 2579 (48.8) | 3388 (44.1) |  | 1867 (47.5) | 1843 (46.9) |  |
|  | **III** | 1670 (31.6) | 3283 (42.7) |  | 1397 (35.5) | 1432 (36.4) |  |
| **T Stage** | **T1** | 2764 (52.3) | 3258 (42.4) | <0.001 | 1870 (47.6) | 1862 (47.4) | 0.983 |
|  | **T2** | 2094 (39.6) | 3699 (48.2) |  | 1730 (44.0) | 1735 (44.1) |  |
|  | **T3** | 187 (3.5) | 408 (5.3) |  | 159 (4.0) | 165 (4.2) |  |
|  | **T4** | 237 (4.5) | 316 (4.1) |  | 172 (4.4) | 169 (4.3) |  |
| **The Number of Positive LN** | **1** | 4188 (79.3) | 4182 (54.4) | <0.001 | 2877 (73.2) | 2842 (72.3) | 0.293 |
|  | **2** | 852 (16.1) | 2189 (28.5) |  | 813 (20.7) | 814 (20.7) |  |
|  | **3** | 242 (4.6) | 1310 (17.1) |  | 241 (6.1) | 275 (7.0) |  |
| **Type of Surgery** | **No/Unknown** | 153 (2.9) | 4 (0.1) | <0.001 | 2 (0.1) | 4 (0.1) | 0.267 |
|  | **BCS** | 3309 (62.6) | 3071 (40.0) |  | 2214 (56.3) | 2151 (54.7) |  |
|  | **Mastectomy** | 1820 (34.5) | 4606 (60.0) |  | 1715 (43.6) | 1776 (45.2) |  |
| **Radiation** | **Yes** | 2870 (54.3) | 3198 (41.6) | <0.001 | 1992 (50.7) | 1971 (50.1) | 0.652 |
|  | **No/Refused** | 2412 (45.7) | 4483 (58.4) |  | 1939 (49.3) | 1960 (49.9) |  |
| **Chemotherapy** | **Yes** | 1301 (24.6) | 2783 (36.2) | <0.001 | 1115 (28.4) | 1137 (28.9) | 0.600 |
|  | **No/Unknown** | 3981 (75.4) | 4898 (63.8) |  | 2816 (71.6) | 2794 (71.1) |  |
| **ER Status** | **Positive** | 4594 (87.0) | 6099 (79.4) | <0.001 | 3329 (84.7) | 3309 (84.2) | 0.554 |
|  | **Negative** | 688 (13.0) | 1582 (20.6) |  | 602 (15.3) | 622 (15.8) |  |
| **PR Status** | **Positive** | 3983 (75.4) | 5137 (66.9) | <0.001 | 2844 (72.3) | 2833 (72.1) | 0.801 |
|  | **Negative** | 1299 (24.6) | 2544 (33.1) |  | 1087 (27.7) | 1098 (27.9) |  |
| **HER2 Status** | **Positive** | 447 (8.5) | 651 (8.5) | <0.001 | 341 (8.7) | 369 (9.4) | 0.490 |
|  | **Negative** | 3174 (60.1) | 3071 (40.0) |  | 2008 (51.1) | 1953 (49.7) |  |
|  | **Borderline** | 85 (1.6) | 110 (1.4) |  | 60 (1.5) | 55 (1.4) |  |
|  | **Not 2010+ Breast** | 1576 (29.8) | 3849 (50.1) |  | 1522 (38.7) | 1554 (39.5) |  |
