# Supplementary material for: Association of Axillary Lymph Node Evaluation With Survival in Women Aged 70 Years or Older With Breast Cancer
Source: Front Oncol. 2021 Jan 28;10:596545. doi: 10.3389/fonc.2020.596545 (PMC7877252; doi:10.3389/fonc.2020.596545)
Supplement: Supplementary file 7 [file Table_4.doc]

**Supplemental Table 4.** Baseline characteristics of hormone receptor positive (HR+) and hormone receptor negative (HR-) subgroups in matching pN1 stage patients

| **Characteristics** | **Hormone Receptor Positive (HR+)** | | | **Hormone Receptor Negative (HR-)** | | |
| --- | --- | --- | --- | --- | --- | --- |
|  | **SLNB** | **ALND** | ***P* value** | **SLNB** | **ALND** | ***P* value** |
| **N** | 3358 | 3335 |  | 573 | 596 |  |
| **Age** |  |  | 0.144 |  |  | 0.048 |
| 70-74 | 1304(38.8) | 1224(36.7) |  | 180(31.4) | 231 (38.8) |  |
| 75-79 | 925(27.5) | 985(29.5) |  | 154(26.9) | 154 (25.8) |  |
| 80-84 | 678(20.2) | 652(19.6) |  | 125(21.8) | 105 (17.6) |  |
| 85+ | 451(13.4) | 474(14.2) |  | 114(19.9) | 106 (17.8) |  |
| **Race** |  |  | 0.486 |  |  | 0.282 |
| White | 2851(84.9) | 2864(85.9) |  | 466(81.3) | 469(78.7) |  |
| Black | 280(8.3) | 254(7.6) |  | 72(12.6) | 94(15.8) |  |
| Other | 227(6.8) | 217(6.5) |  | 35(6.1) | 33(5.5) |  |
| **Marital** |  |  | 0.910 |  |  | 0.012 |
| Married | 1411(42.0) | 1395(41.8) |  | 198(34.6) | 256(43.0) |  |
| Single | 1812(54.0) | 1799(53.9) |  | 346(60.4) | 317(53.2) |  |
| Unknown | 135(4.0) | 141(4.2) |  | 29(5.1) | 23(3.9) |  |
| **Laterality** |  |  | 0.343 |  |  | 0.675 |
| Right | 1655(49.3) | 1604(48.1) |  | 198(34.6) | 256(43.0) |  |
| Left | 1703(50.7) | 1731(51.9) |  | 346(60.4) | 317(53.2) |  |
| **Grade** |  |  | 0.630 |  |  | 0.133 |
| I | 653(19.4) | 649 (19.5) |  | 14(2.4) | 7(1.2) |  |
| II | 1762(52.5) | 1716 (51.5) |  | 105(18.3) | 127(21.3) |  |
| III | 943(28.1) | 970 (29.1) |  | 454(79.2) | 462(77.5) |  |
| **T Stage** |  |  | 0.862 |  |  | 0.147 |
| T1 | 1653(49.2) | 1661(49.8) |  | 217(37.9) | 201(33.7) |  |
| T2 | 1462(43.5) | 1422(42.6) |  | 268(46.8) | 313(52.5) |  |
| T3 | 119(3.5) | 120(3.6) |  | 40(7.0) | 45(7.6) |  |
| T4 | 124(3.7) | 132(4.0) |  | 48(8.4 | 37(6.2) |  |
| **Type of Surgery** |  |  | 0.061 |  |  | 0.296 |
| No | 1(0.0) | 3(0.1) |  | 1(0.2) | 1(0.2) |  |
| BCS ^d^ | 1945(57.9) | 1844(55.3) |  | 269(46.9) | 307(51.5) |  |
| Mastectomy | 1412(42.0) | 1488(44.6) |  | 303(52.9) | 288(48.3) |  |
| **Radiation** |  |  | 0.238 |  |  | 0.094 |
| Yes | 1745(52.0) | 1684(50.5) |  | 247(43.1) | 287(48.2) |  |
| No/Refused | 1613(48.0) | 1651(49.5) |  | 326(56.9) | 309(51.8) |  |
| **Chemotherapy** |  |  | 0.683 |  |  | 0.079 |
| Yes | 831(24.7) | 810(24.3) |  | 284(49.6) | 327(54.9) |  |
| No/Unknown | 2527(75.3 | 2525(75.7) |  | 289(50.4) | 269(45.1) |  |
| **ER Status** |  |  | 0.806 |  |  | NA |
| Positive | 3329(99.1) | 3309(99.2) |  | 0 | 0 |  |
| Negative | 29(0.9) | 26(0.8) |  | 573(100.0) | 596(100.0) |  |
| **PR Status** |  |  | 0.798 |  |  | NA |
| Positive | 2844(84.7) | 2833(84.9) |  | 0(0.0) | 0(0.0) |  |
| Negative | 514(5.3) | 502(15.1) |  | 573(100.0) | 596(100.0) |  |
| **HER2 Status** |  |  | 0.663 |  |  | 0.734 |
| Positive | 248(7.4) | 269(8.1) |  | 93(16.2) | 100(16.8) |  |
| Negative | 1767(52.6) | 1718(51.5) |  | 241(42.1) | 235(39.4) |  |
| Borderline | 49(1.5) | 46(1.4) |  | 11(1.9) | 9(1.5) |  |
| Not 2010+ | 1294(38.5) | 1302 (39.0) |  | 228(39.8) | 252(42.3) |  |

*NA* not applicable
